# Companion robots to mitigate loneliness among older adults: Perceptions of benefit and possible deception

**DOI:** 10.3389/fpsyg.2023.1106633

**Published:** 2023-02-21

**Authors:** Clara Berridge, Yuanjin Zhou, Julie M. Robillard, Jeffrey Kaye

**Affiliations:** ^1^School of Social Work, University of Washington, Seattle, WA, United States; ^2^Steve Hicks School of Social Work, University of Texas at Austin, Austin, TX, United States; ^3^Faculty of Medicine, University of British Columbia, Vancouver, BC, Canada; ^4^School of Medicine, Oregon Health and Science University, Portland, OR, United States

**Keywords:** robotics, artificial intelligence, natural language processesing, dementia, ethics

## Abstract

**Objective:**

Given growing interest in companion robots to mitigate loneliness, large-scale studies are needed to understand peoples’ perspectives on the use of robots to combat loneliness and attendant ethical issues. This study examines opinions about artificial companion (AC) robots regarding deception with dementia and impact on loneliness.

**Methods:**

Data are from a survey of 825 members of the OHSU Research via Internet Technology and Experience cohort (response rate = 45%). Sixty percent (*n* = 496) of the age diverse sample (range = 25–88; *M* = 64; SD = 13.17) is over 64, allowing us to compare across age and consider current and future older adults. Ordinal logistic regressions examined relationships between age, health, and other socio-demographic characteristics and perceptions of impact on loneliness and comfort with deception.

**Results:**

Most participants (68.7%) did not think an AC robot would make them feel less lonely and felt somewhat-to-very uncomfortable (69.3%) with the idea of being allowed to believe that an artificial companion is human. In adjusted models, one additional year of age was associated with lower likelihood of perceived benefit of reducing loneliness [Odds Ratio (OR) = 0.98; (0.97–0.99), *p* = 0.003] and lower comfort with deception [OR = 0.99; (0.97–1.00), *p* = 0.044]. Being female was associated with lower likelihood of comfort with deception [OR = 0.68; (0.50–0.93), *p* = 0.014] and high confidence using computers with greater comfort [OR = 2.18; (1.42–3.38), *p* < 0.001].

**Discussion:**

There was not strong support for AC robots to mitigate loneliness. Most participants were uncomfortable with this form of deception, indicating need for design solutions for those who want to avoid this possibility, as well as greater attentiveness to desirability and comfort across age and gender.

## 1. Introduction

Social isolation among older adults during the coronavirus disease 2019 (COVID-19) pandemic has been termed “the double pandemic” (Holt-Lunstad, 2020). Attention is growing on new ways to mitigate loneliness for older adults and specifically people living with dementia, spurred by the increased risk for loneliness among older adults due to the pandemic (Tam et al., 2021) and findings that dementia increases risk for loneliness (Sutin et al., 2020). In the context of social isolation and inadequate resources to meet elder care needs, artificial companion robots - devices that use AI to interact conversationally - have been developed to keep older adults company, among other functions (Jackson, 2019; Portacolone et al., 2020; Berridge et al., 2021; Coghlan et al., 2021; Engelhart, 2021; Sekhon et al., 2022). This is a topic increasingly relevant to aging services. In the United States, state aging departments have distributed AI-based robots to older adults in response to the challenges of meeting the socialization needs of isolated older adults during the pandemic (Zilber, 2022). The use of robots with older adults had received media attention prior to COVID, but this intensified during the pandemic (Jackson, 2019; Samuel, 2020). For example, a New Yorker article reported that a number of states started robot programs, some paid for by pandemic-relief funding, and that aging departments in 21 states have distributed more than 20,000 furry robot pets expressly to help lonely older people (Engelhart, 2021).

Most of the research has focused on pet-like robots that do not have natural language processing capability (Sekhon et al., 2022). A systematic review of 11 studies that examined non-speaking, primarily plush pet-like robots used with older adults living with dementia found that they have the potential to improve quality of life, agitation and anxiety, engagement and social interaction, loneliness, stress, and medication use, though the review determined the studies to be of low to moderate quality (Pu et al., 2019). Telepresence and non-pet-like robots have been successfully piloted in residential facilities with people living with dementia to serve as platforms for arts-based interventions (Fields et al., 2021). Small pilots with older adults living with dementia and/or depression suggest feasibility of AI-conversational robots (Abdollahi et al., 2017; Khosla et al., 2021); however, there is very little evidence that speaking, artificially intelligent companions either mitigate or contribute to social isolation or loneliness (Robillard et al., 2020).

A cross-sectional study of the effects of COVID-19 on perception of and intention to purchase a social robot found that loneliness was positively associated with reported willingness to buy a robotic companion (Ghafurian et al., 2021), indicating that people may perceive that a robotic companion could mitigate loneliness. Similarly, a study that predates the pandemic of non-AI robots, Paro and Giraff (telepresence), suggests a role for psychosocial functioning (depressive mood, loneliness, life satisfaction and social support) in robot acceptance among older adults (Baisch et al., 2017). A small study of robots use in dementia care found that participants were concerned that it could increase isolation for this group (Natarajan et al., 2022). However, the research that assesses opinions of potential users about AI companions’ proposed benefit of mitigating loneliness is very limited, and small-scale studies cannot assess potential differences across groups.

The ethical issues related to the surveillance that artificial companions enable, deception, the potential for reduced opportunities for human interaction, and the difficulty achieving informed consent of people living with dementia are open topics of interest in academic journals (Vandemeulebroucke et al., 2018; Portacolone et al., 2020; Robillard et al., 2020). In a study on the risks and benefits of dementia care technologies in the U.S and Canada, a number of domain expert participants used the word “problematic” in reference to using AI for companionship (Berridge et al., 2021). Reported potential risks of using companion robots include reduced human interaction, increased isolation, depersonalization in robot relationships, frustration for people living with dementia caused by errors, confusion about where the voice is coming from, overreliance, and the risk of depriving people of meaningful connection (Berridge et al., 2021).

Portacolone et al. (2020) have argued that a core ethical problem is deception when older adults believe they are in a personal relationship with an artificial companion robot. Van Wynsberghe (2022) pinpoint the ethical problem as one of deception that a robot is deserving of reciprocity, which is enabled through the form and responsive capabilities designed into it. The possibility of deception, particularly when dementia is present, has received attention both in the popular media and the academic literature. Itis a particularly compelling challenge.

Vandemeulebroucke et al. (2018) suggest that all stakeholders in aging services should have a voice in the discussion to complement ethical assessments and ethical reflection. Robillard et al. (2020) have specifically called for more empirical research on the attitudes of older adults toward deception with fully automated robotics that seem human-like or human-controlled to inform the ethical debate. They explain why preventing deception of people living with dementia may not be as simple as controlling form design, and they argue that efforts to do so to prevent harm should be informed by stronger evidence of possible harm (Robillard et al., 2020). Deception in the form of mismatch between appearance and source (i.e., AI with human voice in pet-like form or a human remotely speaking and visually represented vs. represented by an animal avatar) is not the only form of deception. Robillard and colleagues cite evidence that emotionally responsive assistive technologies for older adults may be more effective than those without affect expression capability and point out that “people have a strong tendency to read human-like intent into many different types of technological artifacts” (Robillard et al., 2020). Leong and Selinger (2019) build on the principle of “honest anthropomorphism” (see Kaminski et al., 2017) with a taxonomy of forms of what they term “dishonest anthropomorphism” to which humans are inherently vulnerable (Leong and Selinger, 2019). This refers to misalignments between the capabilities of a robot and the assumptions a person makes about that robot’s capabilities. This misalignment takes many forms, such as in human responses to the particular voice chosen for the robot and expression by the robot of non-existent emotion, opinion or attitudes. These issues are heightened by very recent developments such as Amazon’s Alexa’s voice assistant’s new demo feature of recreating human voices from audio clippings, including those of deceased individuals (Paul, 2022). Amazon’s stated goal of this feature was “to build greater trust with users by infusing artificial intelligence with the human attributes of empathy and affect” Rohit Prasad as cited by Paul (2022). Leong and Selinger’s (2019) taxonomy of “dishonest anthropomorphism” raises complex questions that are increasingly relevant to real-world decisions, such as how do these misalignments promote inappropriate levels of trust? These open issues have important implications for privacy, autonomy and boundary management (Berridge, 2016; Leong and Selinger, 2019).

The current study examines among a large online cohort of adults in the United States, opinions about the potential of AC robots to mitigate loneliness and comfort with possible deception with their use in the context of dementia. We further examine how these opinions and comfort levels vary by key socio-demographic characteristics. This article reports on whether respondents think an AC robot would help address loneliness for them, as well as how they feel about deception with dementia, should they believe that the voice of an AC is a real human. We analyze free text comments on the survey that provide nuance and further insight to a range of feelings people express about AC robots.

## 2. Methods

### 2.1. Study design and population

The 19-item survey that we report data from was administered using Qualtrics and disseminated by email in June of 2020 to the online survey cohort of the Research via Internet Technology and Experience (RITE) program of the Oregon Center for Aging & Technology (ORCATECH) at Oregon Health & Science University (OHSU) University. Volunteers in this cohort are adults who complete topical surveys quarterly about technology and health and wellness. The RITE online cohort was launched in 2015 to identify and track attitudes and preferences of technology use in healthcare over time. The current study used the full sample of 2,434 volunteers registered as active in 2019. The RITE cohort had no inclusion criteria other than being over the age of 18. Volunteers were primarily recruited through direct email invitations using OHSU’s Oregon Clinical and Translational Research Institute’s (OCTRI) Cohort Discovery, which interfaces with OHSU’s EPIC electronic medical record data repository maintained by OCTRI. Social media campaigns and flyers were secondary recruitment strategies. RITE volunteers completed online an initial packet immediately after consent (OHSU IRB # IRB00010237) and an annual online survey to report changes to the information gathered in their initial packet. The full cohort of 2,434 members was sent the online survey and 1,082 completed it (response rate = 45%).

Two respondents were not living in the community and were thus excluded, as were those without data for four core variables: gender (missing = 72), age (missing = 4), education (missing = 150), or memory problem history (missing = 179), leaving an analytic sample of 825 respondents. The rate of missing value for each of the other covariates were each below 10% (0.1–9.1% for the variable of history of dementia in parents). Gender was recorded in the initial intake for the RITE cohort as a binary response option of male and female and a write-in option. We coded those who wrote in transgender man or woman with male and female and excluded for analysis the six people whose written-in responses fell broadly under categories such as gender diverse and questioning, discussed further in the limitations section. Because we omitted from our sample the 16% of participants who had missing values for the key variable of interest, reported history of memory problems, we conducted sensitivity analyzes stratifying by each outcome.

### 2.2. Dependent variables

The survey introduced companion robots in the following way: “Interest is growing in artificial intelligence that is built into robots. Robots can be made to look like animals or humans. One use for these robots is to provide companionship because these robots can hold conversations with people.” To make this concrete for participants, two example images were provided: one of the products called GenieConnect and one of ElliQ. Participants were asked, “If you were feeling lonely, do you think that an artificial companion that can talk with you would make you feel less lonely?” (Definitely No, Probably No, Probably Yes, and Definitely Yes), and “If you had dementia, how comfortable would you be with your primary support person letting you believe that an artificial companion is a real human?” (Very Uncomfortable, Somewhat Uncomfortable, Somewhat Comfortable, Very Comfortable). Each response option was labeled for consistent interpretation. Please see Supplementary Material for these survey questions. Participants were also provided with an open response comment option at the conclusion of the survey with the prompt, “Do you have any comments you’d like to share?”

### 2.3. Independent variables

Health and demographic information was pre-collected through the RITE cohort surveys. Characteristics previously associated with comfort and preferences for digital technologies were included in analyzes, including memory problem history (Charness and Boot, 2009), which is a yes response if answered yes to one of two questions about (1) presence of self-reported current memory problems or (2) if the participant has been seen by a physician for memory problems. We included age (Thordardottir et al., 2019), gender (Lai et al., 2010; Gell et al., 2015), marital status (Gell et al., 2015; Abd-Alrazaq et al., 2019), living status (Lai et al., 2010), education (Lai et al., 2010; Gell et al., 2015), number of chronic conditions (Chappell and Zimmer, 1999; Lai et al., 2010), confidence of using computer (Czaja et al., 2006), and social support (Baisch et al., 2017) defined as level of social activity using the Brief Assessment of Social Engagement scale (0–20) (Morgan et al., 1985). Because we are interested in examining potential differences by memory status, we included memory problem history, as well as history of dementia in parents because these might indicate respondents’ perceived risk of acquiring dementia (Kessler et al., 2012) and because the perspective gained about dementia may be influential on these questions of interest. We also included pet ownership because that experience might impact one’s feelings about living and interacting with a non-human companion, such as a small robot. There is insufficient variability for analysis by race and ethnicity: 95.9% of respondents were white and 98.5% were non-Hispanic, discussed further in the limitations section.

### 2.4. Analysis

Analyzes were performed using R software (R Core Team, 2013). Bivariate and multivariate ordered logistic regressions (Bilder and Loughlin, 2014) were performed using the R package “MASS” (Ripley, 2011) and “ordinal” (Christensen and Christensen, 2015) to determine whether there were relationships between independent variables and dependent variables that are ordinal (Long and Freese, 2006). Brant tests were used to test the assumption of proportional odds (UCLA, Statistical Consulting Group, n.d.). To better understand how different critical factors drive the specific trends, we conducted post-hoc interaction analysis on variables that are significantly associated with outcome variables in bivariate and multivariate analysis. As shown in the Supplementary Material Table, for both outcomes, we examined possible interactions between age and education, education and memory problem history, and gender and memory problem history.

After completing the survey questions about AC robots, participants were asked to provide their comments in an open text box. Thematic analysis was conducted on these qualitative responses provided by 315 participants (38%) (Nowell et al., 2017). Two members of the research team read all the responses and separately developed initial codebooks. They met to merge their codes into a single codebook and to refine it. They then separately coded the comments and met to discuss all discrepancies where codes were differently applied until they reached consensus about final coding (Nowell et al., 2017). Seven themes were identified that relate to the issues of loneliness mitigation and deception. Below, we present frequencies for prominent themes along with exemplary comments.

## 3. Results

### 3.1. Participant characteristics

Table 1 presents participant characteristics. Participants ranged in age from 25 to 88, but the sample skewed older with a mean of 64-years-old (SD = 13.17). Sixty-five percent identified as female and 35% as male and 70% were married or living as if married, while 20% lived alone. One quarter had no college degree, one third had a college degree, and 42% had a master’s degree or more. About one quarter reported having memory problems and 68% had 3 or more chronic conditions. Thirty percent had a parent with a history of dementia. The majority (84%) were highly confident using computers, with only about 16% reporting moderate to low confidence. Sixty-two percent reported interacting often with a pet and the sample’s mean social activity score was 8.47 (range = 0–17; SD = 2.82) with a maximum possible of 20.

**Table 1:** Participant characteristics.

Category	Subcategories	Mean/SD/frequencies	Percentage (%)
Age (*n* = 825)	Range: 25–88	Mean = 63.93 SD = 13.17	
Gender (*n* = 825)	Female	534	64.7
	Male	291	35.3
Race/ethnicity (*n* = 819)	Non-Hispanic white	776	94.7
	Non-Hispanic Black	6	0.7
	Hispanic	11	1.3
	American Indian or Alaska Native	5	0.6
	Asian	10	1.2
	Others	11	1.3
Marital status (*n* = 820)	Married/living as if married	577	70.4
	Not married	243	29.6
Living status (*n* = 824)	Living alone	162	19.7
	Living with others	662	80.3
Education (*n* = 825)	No college degree	202	24.5
	College degree	276	33.5
	Master’s degree and above	347	42.1
Memory problem history (*n* = 825)	Memory problem reported	201	24.4
	No memory problem reported	624	75.6
Number of chronic conditions (*n* = 790)	3+	540	68.4
	0–2	250	31.6
Confidence using computer (*n* = 792)	Highly confident	668	84.3
	Low-moderately confident	124	15.7
History of dementia in either parent (*n* = 750)	Yes	226	30.1
	No	524	69.9
Interaction with pet (*n* = 812)	Often interact with pet (daily, weekly, monthly)	503	61.9
	Do not often interact with pet (yearly, rarely, or never)	309	38.1
Social activity level score (*n* = 800)	Range: 0–17 (out of 20)	Mean = 8.47 SD = 2.82	

### 3.2. Survey responses

Most participants did not think an artificial companion that can talk would make them feel less lonely. As depicted in Table 2, those older than 64 were even less likely than their younger counterparts to think it would help with loneliness. One quarter (25.3%) of the full sample definitely did not, while only 3.2% definitely did. 43.4% responded that it probably would not and 28% thought it probably would make them feel less lonely. Most participants were either very uncomfortable (43.6%) or uncomfortable (25.7%) with their primary support person letting them believe that an artificial companion is a real human if they had dementia. About one fifth (21.9%) were somewhat comfortable and 8.8% were very comfortable with this. Respondents over age 64 were less comfortable than were younger respondents with this form of deception (see Table 2).

**Table 2:** Response frequencies, *n* = 825.

If you were feeling lonely, do you think that an artificial companion that can talk with you would make you feel less lonely?				
	Definitely no *n* (%)	Probably no *n* (%)	Probably yes *n* (%)	Definitely yes *n* (%)
65 +	137 (28.4)	231 (47.8)	103 (21.3)	12 (2.5)
<65	67 (20.7)	119 (36.8)	123 (38.1)	14 (4.3)
Total sample	204 (25.3)	350 (43.4)	226 (28.0)	26 (3.2)

If you had dementia, how comfortable would you be with your primary support person letting you believe that an artificial companion is a real human?				
	Very uncomfortable *n* (%)	Somewhat uncomfortable *n* (%)	Somewhat comfortable *n* (%)	Very comfortable *n* (%)
65 +	235 (48.9)	116 (24.1)	97 (20.2)	33 (6.9)
<65	115 (35.7)	90 (28.0)	79 (24.5)	38 (11.8)
Total sample	350 (43.6)	206 (25.7)	176 (21.9)	71 (8.8)

In bivariate analysis, each year of greater age was associated with lower likelihood of believing that AC robots would reduce loneliness [OR = 0.98 (0.97–0.99), *p* < 0.001]. Those with a master’s degree or higher were also less likely to perceive the benefit of AC robots in reducing loneliness [OR = 0.69, (0.50,0.96), *p* = 0.026], as were those with 3+ chronic conditions compared with those with fewer than 3 [OR = 0.63 (0.47–0.83), *p* = 0.001]. Participants reporting a history of memory problems were more likely to perceive this benefit [OR = 1.37 (1.01–1.85), *p* = 0.043].

Higher age [OR = 0.99 (0.97–0.99), *p* < 0.001] and greater number of chronic conditions [OR = 0.64 (0.48–0.84), *p* = 0.002] were also negatively associated with comfort with deception. Greater computer confidence was associated with greater comfort with deception [OR = 2.26 (1.54–3.36), *p* < 0.001] (Table 3).

**Table 3:** Bivariate and multivariate ordinal logistic regression.

	Perceived benefit of AC robots reducing loneliness		Comfort with deception	
	Unadjusted OR (95% CI)	Adjusted OR (95% CI)	Unadjusted OR (95% CI)	Adjusted OR (95% CI)
Age	0.98***	0.98**	0.99***	0.99*
	(0.97–0.99)	(0.97–0.99)	(0.97–0.99)	(0.97–1.00)
Female (vs. Male)	1.10	1.00	0.78	0.68*
	(0.84–1.43)	(0.73–1.36)	(0.61–1.00)	(0.50–0.93)
Married/living as if married (vs. Not married)	0.85	0.83	1.10	1.17
	(0.64–1.13)	(0.51–1.34)	(0.83–1.46)	(0.72–1.90)
Living alone (vs. Living with others)	0.99	0.98	0.81	1.09
	(0.71–1.37)	(0.55–1.74)	(0.58–1.11)	(0.62–1.94)
College degree (vs. No college degree)	0.74	0.87	1.01	1.15
	(0.53–1.04)	(0.59–1.27)	(0.72–1.42)	(0.78–1.71)
Master’s degree and above (vs. No college degree)	0.69*	0.76	1.20	1.25
	(0.50–0.96)	(0.52–1.11)	(0.86–1.67)	(0.85–1.83)
Memory problem history (vs. No history reported)	1.37*	1.38	1.17	1.15
	(1.01–1.85)	(0.99–1.94)	(0.87–1.57)	(0.82–1.62)
3+ chronic conditions (vs. 0–2)	0.63**	0.79	0.64**	0.78
	(0.47–0.83)	(0.56–1.10)	(0.48–0.84)	(0.56–1.10)
High confidence in using computers (vs. Low-moderately confidence)	1.35	1.16	2.26***	2.18***
	(0.94–1.93)	(0.78–1.73)	(1.54–3.36)	(1.42–3.38)
History of dementia in parents (vs. No history of dementia in either of parents)	0.76	0.95	1.05	1.19
	(0.56–1.01)	(0.70–1.31)	(0.78–1.40)	(0.87–1.63)
Often interact with pet (vs. Not often interact with pet)	1.06	0.85	1.10	1.00
	(0.82–1.38)	(0.63–1.16)	(0.84–1.43)	(0.74–1.36)
Social activity level score	0.97	1.00	1.01	1.03
	(0.93–1.02)	(0.95–1.06)	(0.97–1.06)	(0.98–1.09)

*p < 0.05; **p < 0.01; ***p < 0.001.

In multivariate analysis, unlike in bivariate analysis, those with a history of memory problems, 3+ chronic conditions, and those with master’s degrees and higher were no different from their counterparts in their perception of AC robot potential to help them feel less lonely. Greater age continued to be negatively associated with perceived benefits of AC reducing loneliness [OR = 0.98, (0.97,0.99), *p* = 0.003], and deception related to AC [OR = 0.99; (0.97, 1.00), *p* = 0.044]. This means that with each 1 year of additional age, people have a 2% lower likelihood of believing that AI will reduce loneliness for each level (definitely no versus probably no, probably no versus probably yes, and probably yes vs. definitely yes), controlling for other variables. One additional year of age is associated with a 1% lower likelihood of being comfortable with deception; that is, to report very comfortable versus somewhat comfortable, somewhat comfortable versus somewhat uncomfortable, and somewhat uncomfortable versus very uncomfortable. In multivariate analysis, being female vs. male was associated with lower comfort with deception [OR = 0.68, (0.50–0.93), *p* = 0.014]. As with our bivariate analysis, controlling for other factors, people reporting high confidence using the computer were more than twice as likely to report greater comfort with AC deception [OR = 2.18; (1.42, 3.38), *p* < 0.001].

Significant interaction effects were found among education, age, gender, and memory problem history. There was an interaction effect of age and education level on participants’ perceived benefits of AC robots in reducing loneliness (Supplementary Table S1; Model 1). Only among participants who had a master’s degree was age significantly associated with a lower likelihood of perceiving the benefits of AC robots in reducing loneliness (master’s degree: OR=0.96, [0.95,0.98], *p*<0.000; college degree: OR=0.99, [0.97,1.01], *p*=0.210; no college degree: OR=0.99, [0.97,1.02], *p*=0.625). There was also an interaction effect of gender and memory problem history on participants’ perceived benefits of AC robots in reducing loneliness (Supplementary Table S1; Model 2). For male participants, there were no significant associations between memory problem history and this perceived benefit (OR=0.84, [0.48,1.48]. *p*=0.542). However, among female participants, having a memory problem history was significantly associated with a higher likelihood of perceiving that AC robots could reduce loneliness. Only among participants who had no college degree was having a memory problem history significantly associated with a higher likelihood of perceiving the benefits of AC robots in reducing loneliness (no college degree: OR=2.72, [1.36, 5.41], p=0.005; college degree: OR=1.23, [0.70, 2.17], p=0.474; master’s degree: OR=1.02, [0.60,1.74]. p=0.949).

### 3.3. AC robots in participants’ own words

Themes derived from comments offered by 38% of the participants provide insights into their feelings about this use of AC robots. The most commonly raised issue (*n* = 45) was regarding the invasion of privacy and perception that AC robots that rely on and collect audio data constitute over monitoring. This issue was often coupled with statements about data security, third party use, and possible data exploitation as unresolved problems that were cause for concern. Another common theme in the comments (32) was that human experiences cannot or should not be replaced, with concern over potential loss of real human interactions, meaning, affection, empathy and compassion. This participant’s comment echoes a common sentiment: “One of the problems I see with how we care for the elderly is the lack of contact with others. I am afraid that these measures would lead to less and less human contact for these folks. It might become easier and cheaper for the care system to use these measures and for our elderly to become more and more isolated.” Others (15) acknowledged positive potential uses of AI-enabled robots to assist with physical tasks and drew a line at social interaction: “After using Google Home (a very simple robot), I am familiar with talking to ‘technology’ and have no problem using it to control things around my home. However, social interaction is a different thing, and although I think I know how I would feel about having a tech buddy, I’m not sure how I would feel if I actually interacted with one. Being a retired techie, I use a lot of tech to make my life easier and try to stay up to date, so I do not have an aversion to using it but feel that human to human interaction is also very important.” Some participants (22) specified that they would prefer to have a human or a pet over an AC robot.

Twenty-three people focused their comments on ethical problems, including but not limited to the issue of deception, or they wrote that AC robots are troubling, disturbing, dangerous, or a slippery slope that would undermine care. As one explained, “All of these artificial companions provide the illusion of intimacy without actual intimacy. That’s dishonest - and creepy.” Like a small number of others, this participant offered that AC robots are the wrong solution to the problem: “The answers to the problems implicit in these prompts cannot be found on robots - they can only be found in the difficult, and necessary, work of restructuring our society so that people who need it always have in-person support.” Another suggested, “We need to temper AI with HI-Human Intelligence systems that are financially supported and that provide healthy human interactions rather than pretending that Alexa is your ‘friend.’ That is AI jail keeping, not community building.”

Another common theme was not being able to project how one would feel if they acquired dementia, or that it would depend on a number of health and functioning realities. For example, a participant wrote, “I think that the answers to several of these questions would be different depending on whether I could talk, my level of dementia, and other factors. It is hard to decide in a vacuum.” This idea that decisions about AC should be contextualized was commonly noted. Another 16 projected that if they had dementia, they would not have an opinion or care about how AC robots were used with them.

## 4. Discussion

This survey research conducted with an online community with a variety of health and socio-demographic factors provided a unique opportunity to learn about the perceived impact of AC robots on loneliness and the level of comfort with deceiving people with dementia that the talking robot is not human. Aligned with much of the ethics conversations about deception, most participants were uncomfortable with their primary support person letting them believe that an AC is a real human if they had dementia.

People reporting a history of memory problems or history of a parent with dementia were no more likely to be comfortable or uncomfortable with this form of deception. This is an interesting finding that is consistent with a very small body of research suggesting little to no difference in comfort with monitoring technology or data collection according to mild cognitive impairment status (Boise et al., 2013). It indicates that despite perceived vulnerability, countervailing factors may be at play for those who are concerned about potential memory difficulties, perhaps including consideration for dignity, autonomy loss, or desire for control (Mcdonald and Mentis, 2021); however, this is only speculative and requires further research to understand the considerations underlying this finding of no difference. This is a particularly important area for future research because companion robots are developed for use with people living with dementia with potential benefits of stimulating cognitive engagement, enabling people to use their language functions, as well as experience interactions that are free from human responses of impatience with repetition. However, domain experts and ethicists caution that the use of AC robots could deceive and confuse the person living with dementia about where the voice is coming from, as well as potentially deprive people of meaningful conversation and lead to depersonalization (Berridge et al., 2021). This study’s participants, the majority of whom were over the age of 64, provides important insights into how potential users feel about this ethical issue; however, while many reported experiencing memory issues, these participants were not living with dementia. In comments, some participants described their difficulty projecting out how they would feel about deception should they acquire dementia, noting that it would depend on numerous factors and the decision should be contextualized. It is thus critical that people who are living with mild cognitive impairment (MCI) and dementia be included in these conversations. Our findings also indicate that it will be important to examine potential differences by gender among older adults with MCI and dementia, including explanations for what impacts those differences, and how to address them accordingly (i.e., through product design and practices).

In bivariate analysis, higher age and greater number of chronic conditions were associated with lower comfort with this form of deception, while greater computer confidence was associated with greater comfort with it. Controlling for other variables, higher age and identifying as female (vs. male) were each associated with lower comfort, while high confidence using computers remained associated with greater comfort. We found a positive relationship between high confidence using computers and comfort with being allowed to believe, that an AC robot is a human. It is possible that this indicates that greater trust or reliance on computers may accompany higher feelings of mastery or competence in relation to other digital technologies. This requires more systematic analysis than our reported comments provided of the reasons people feel comfort or discomfort with this form of deception.

These findings regarding age and gender are consistent with other research on comfort with data collection and sharing generally, which reports those who identify as female are less comfortable than are males with various types of data collection about them, potentially due to greater risks or sense of vulnerability to online abuses or exposures (Li, 2011; Matthews et al., 2017; Messing et al., 2020; Berridge et al., 2022). While this form of deception is a different kind of data flow question than personal data sharing preferences, they may both reflect greater weight placed on maintaining a level of control or greater perceived vulnerability to consequences of lacking control. This difference was not assessed qualitatively, so we can only speculate. It should be more closely examined to understand why and how interventions could be responsive to the concerns and needs of female-identified older adults, as well as people with gender identities that were not captured in this study (e.g., non-binary). The association of higher age with lower comfort with this form of deception is important to understand in light of the fact that women make up the majority of older adults, and an even greater proportion of those over age 85.

Regarding impact on loneliness, the majority did not perceive that AC robots would make them feel less lonely, and this did not differ in adjusted models if a person had family histories of dementia. We were interested in potential differences between those with and without a reported memory problem history and found that reporting memory problems was associated with perceiving this benefit in bivariate but not multivariate models. Also in bivariate analysis, both higher age and higher education were associated with lower belief that an AC robot would reduce loneliness, as was having 3+ compared with fewer chronic conditions. Only higher age remained associated with this lower perceived benefit in adjusted models.

For this outcome of perceived benefit of reducing loneliness, there were interaction effects of age and education level, gender and memory problem history, and education and memory problem history. The association between greater age and lower perception of this benefit is stronger among those with the highest level of education compared with those with the lowest. For female participants, reporting a memory problem history was associated with greater likelihood of perceiving this benefit than for those without such a history, whereas for male participants, reporting a memory problem history was not significantly associated with their likelihood of perceiving this benefit. Additionally, for those with the lowest level of formal education, reporting a memory problem history was significantly associated with greater likelihood of perceiving this benefit than it was for those without such a history; such association was significantly greater than it was for those with the highest level of education. These findings imply that future research should closely examine how these characteristics (gender, age, education and memory status) interrelate and impact desire for AC robots and, if implemented, impact on loneliness.

Participants most often offered comments expressing concerns over privacy invasion, data use, and lack of security of data used by AC robots. These echo concerns raised in the literature about the ethical issues related to surveillance enabled by AC robots (Vandemeulebroucke et al., 2018; Portacolone et al., 2020; Robillard et al., 2020), as well as calls for regulation to address data use (Berridge et al., 2021). Free form comments also provide insight into survey findings to the extent that participants preferred task-oriented robots over companion-purpose robots, though the technological capacities of robotics are not nearly refined enough to realize task completion (Maibaum et al., 2022). Preference was expressed for human or pet companionship. These comments are consistent with other studies that found either rejection of robots that pretend to be companions (Deutsch et al., 2019) or desire for companionship only as a secondary but not primary function (Coghlan et al., 2021).

Many of the optional comments further expressed the belief that robots cannot or should not substitute for human care, contact, or touch. Some offered poignant statements about this being the wrong solution to the problem, which they described as deserving of societal restructuring and greater investment in provision of needed in-person supports. Others felt that use of AC robots may further entrench the problem of social isolation among older adults or create the “illusion of intimacy without actual intimacy.” These concerns that align with those raised in the literature suggest that care systems do not become dependent on artificial companionship to attempt to meet needs for human contact, mutuality, and touch. Participants expressed interest in robots that could perform a task or function but were less optimistic that AC robots could provide meaningful support to someone experiencing loneliness. Limitations.

In this study we did not examine whether self-report of loneliness impacts these attitudes toward AC robots. The study sample is 95% white and lacks racial and ethnic diversity, as well as diversity in digital access and literacy, given that this is an online cohort. This sample has above average levels of technological experience as an online cohort and more formal education. As part of intake into the cohort, participants were asked a question about the extent to which their material needs in their adult life have been met. We analyzed our sample’s responses to this question and found too little variation to include it in our models. The vast majority responded that their food, housing, clothing and medical needs have been met. We did not collect income data, but the distribution of this material needs question would suggest that the sample is more financially resourced than the average person in the U.S. Their concerns and preferences may differ from that of the general population. It is also possible that their greater access to digital technologies may make them more aligned in preference and comfort with an early adopter population.

Our measure of memory concerns is derived from two self-reported survey questions. It does not imply a diagnosis of dementia or mild cognitive impairment. The proportion in our sample who reported memory concerns is consistent with population surveys about memory loss concerns (Cooper et al., 2011; Vlachos et al., 2019). Research is also needed with people living with dementia about their perceptions of these issues. We did not oversample those who identify, as six of our respondents did, under the umbrella of gender diverse or questioning, and were thus unable to conduct analysis with this small group. Surveys with more gender diversity representation are needed to better understand and address potential differences by gender, as ours was limited to a binary male/female comparison that does not reflect gender diversity. Finally, perceptions and beliefs may not translate to actual experiences. Nevertheless, these findings provide a snapshot of a non-expert population’s personal ethical assessments of two understudied issues.

## 5. Conclusion

This finding that the majority of respondents did not think an AC robot would help them with loneliness and that this negative appraisal was associated with greater age appears inconsistent with the purported benefit of AC technology for older adults. These findings suggest that greater potential exposure to isolation that older adults face in general might not result in greater acceptance of AC robots to address loneliness. Given the concerns highlighted by a number of participants, it is particularly important that implementation does not get too far ahead of user centered design where older adults, are engaged in the design of interventions so that they are responsive to what older adults want robots to do for them, and policies may then protect the rights and interests of older adult users. Artificial companion robots are targeted on a problem that is not technical in nature (social isolation), so this is particularly important. These responses can inform how we study the impact of social robots and the types of questions we must ask to maximize benefits of robotic and natural language processing capabilities to older adults without reducing human interaction or otherwise causing harm.

## Data availability statement

The datasets presented in this article are not readily available because informed consent was not obtained by participants to share their data with others. Requests to access the datasets should be directed to clarawb@uw.edu.

## Ethics statement

The studies involving human participants were reviewed and approved by University of Washington Human Subjects Division. Written informed consent for participation was not required for this study in accordance with the institutional requirements.

## Author contributions

CB in consultation with JR and JK created the survey. YZ and CB analyzed the data and drafted the manuscript. JR, JK, and YZ edited and reviewed the manuscript. All authors contributed to the article and approved the submitted version.

## Funding

This research was supported by the National Institute on Aging (PI: Berridge, NIA K01AG062681), the Oregon Roybal Center for Care Support Translational Research Advantaged by Integrating Technology (ORCASTRAIT; PI Kaye; supported by NIH P30 AG024978), and the NIA-Oregon Layton Aging and Alzheimer’s Disease Research Center (PI: Kaye, NIA P30AG066518).

## Conflict of interest

The authors declare that the research was conducted in the absence of any commercial or financial relationships that could be construed as a potential conflict of interest.

## Publisher’s note

All claims expressed in this article are solely those of the authors and do not necessarily represent those of their affiliated organizations, or those of the publisher, the editors and the reviewers. Any product that may be evaluated in this article, or claim that may be made by its manufacturer, is not guaranteed or endorsed by the publisher.
