# Supplementary material for: Companion robots to mitigate loneliness among older adults: Perceptions of benefit and possible deception
Source: Front Psychol. 2023 Feb 21;14:1106633. doi: 10.3389/fpsyg.2023.1106633 (PMC9988932; doi:10.3389/fpsyg.2023.1106633)
Supplement: Supplementary file 1 [file Data_Sheet_1.docx]

Supplementary Material

**Companion robots to mitigate loneliness among older adults:
Perceptions of benefit and possible deception**

Clara Berridge,* Yuanjin Zhou, Julie Robillard, Jeffrey Kaye

*** Correspondence:** Corresponding Author: clarawb@uw.edu

# Survey: Survey introduction and questions on companion robots (Q9 for context; Qs 11 and 14 reported)

Intro
**Technology for In-Home Care** These questions ask you about some new technologies used in home care. The questionnaire should take no more than 10 minutes of your time. We are interested in the opinions of people of all ages, regardless of your experience or lack of experience with care. Some of the questions will ask you to think of your primary support person. Your “primary support person” is someone who would be most likely to step in if you needed care or help. We know you may not have a primary support person now but please think about it in terms of your family member or friend who would care for and look out for you.

Start of Block: AI Companionship

Interest is growing in artificial intelligence that is built into robots. Robots can be made to look like animals or humans. One use for these robots is to provide companionship because these robots can hold conversations with people. Please answer the following questions about your comfort with this kind of technology.

[This question provided for context with responses reported elsewhere]

q9_a Please think about unusual times when someone cannot come to your home such as during the coronavirus pandemic. In these times, how comfortable would you be with an artificial companion that can talk with you to keep you company that is in the form of a small robot, like the examples below? [images]

- Very Uncomfortable
- Somewhat Uncomfortable
- Somewhat Comfortable
- Very Comfortable

[This question provided for context with responses reported elsewhere]

q9_b Now please imagine that we are again living under normal circumstances so that you are able to spend time in person with other people.
In normal times, how comfortable would you be with an artificial companion that can talk with you to keep you company that is in the form of a small robot?

- Very Uncomfortable
- Somewhat Uncomfortable
- Somewhat Comfortable
- Very Comfortable

q11 If you were feeling lonely, do think that an artificial companion that can talk with you would make you feel less lonely?

- Definitely No
- Probably No
- Probably Yes
- Definitely Yes

q14 If you had dementia, how comfortable would you be with your primary support person letting you believe that an artificial companion is a real human?

- Very Uncomfortable
- Somewhat Uncomfortable
- Somewhat Comfortable
- Very Comfortable

**2 Supplementary Table 1:** Interaction analysis results

|  | **Perceived benefit of AC robots reducing loneliness** | | |
| --- | --- | --- | --- |
|  | Model 1  Log-odds  (95% CI) | Model 2  Log-odds (95% CI) | Model 3  Log-odds  (95% CI) |
| **Age** | -0.01  (-0.03-0.02) | -0.02 **  (-0.03 - -0.01) | -0.02 **  (-0.03 – -0.01) |
| **Female (vs. Male)** | -0.05  (-0.36-0.26) | -0.18  (-0.53 - 0.17) | -0.01  (-0.32-0.30) |
| **Married/living as if married (vs. Not married)** | -0.21  (-0.70-0.27) | -0.19  (-0.68-0.29) | -0.17  (-0.66-0.31) |
| **Living alone (vs. Living with others)** | -0.05  (-0.62-0.53) | -0.04  (-0.61-0.53) | -0.00  (-0.58-0.57) |
| **College degree (vs. No college degree)** | 0.31  (-1.57-2.18) | -0.10  (-0.48-0.29) | 0.06  (-0.38-0.50) |
| **Master’s degree and above (vs. No college degree)** | 1.70  (-0.12-3.53) | -0.28  (-0.65-0.10) | -0.04  (-0.47-0.38) |
| **Memory problem reported (vs. No memory problem reported)** | 0.34 *  (0.00-0.68) | -0.18  (-0.74-0.39) | 1.00 **  (0.31-1.69) |
| **3+ chronic conditions (vs. 0-2)** | -0.22  (-0.56-0.12) | -0.25  (-0.59-0.09) | -0.26  (-0.06 - 0.08) |
| **High confidence in using computers (vs. Low-moderately confidence)** | 0.18  (-0.22-0.58) | 0.19  (-0.21-0.59) | 0.16  (-0.24-0.56) |
| **History of dementia in parents (vs. No history of dementia in either of parents)** | -0.02  (-0.34-0.30) | -0.04  (-0.35-0.28) | -0.04  (-0.36-0.28) |
| **Often interact with pet (vs. Not often interact with pet)** | -0.17  (-0.47-0.13) | -0.16  (-0.46-0.15) | -0.17  (-0.48-0.13) |
| **Social activity level score** | 0.00  (-0.05-0.06) | 0.00  (-0.05-0.06) | -0.00  (-0.06-0.05) |
| **Age x Education** |  |  |  |
| Age x College degree (vs. No college degree) | -0.01  (-0.04-0.02) |  |  |
| Age x Master’s degree (vs. No college degree) | -0.03 *  (-0.06 - -0.00) |  |  |
| **Gender x Memory problem reported** |  |  |  |
| Female (vs. Male) x Memory problem reported (vs. No memory problem reported) |  | 0.78 *  (0.07-1.49) |  |
| **Memory problem reported x Education** |  |  |  |
| Memory problem reported (vs. No memory problem reported) x College degree (vs. No college degree) |  |  | -0.79  (-1.69-0.10) |
| Memory problem reported (vs. No memory problem reported) x Master’s degree (vs. No college degree) |  |  | -0.98 *  (-1.86 - -0.11) |

*p<0.05; **p<0.01; ***p<0.001

Model 1: Does the association of age and perceived benefits of AC robots in reducing loneliness depend on education level?

Model 2: Does the association of memory problem history and perceived benefits of AC robots in reducing loneliness depend on participants’ gender?

Model 3: Does the association of memory problem history and perceived benefits of AC robots in reducing loneliness depend on education level?
